# Acetabulum fractures in elderly patients: A review

**DOI:** 10.1016/j.cjtee.2021.12.004

**Published:** 2021-12-14

**Authors:** Ashwani Soni, Ravi Gupta, Ramesh Sen

**Affiliations:** a Department of Orthopaedics, Government Medical College and Hospital, Sector-32, Chandigarh, 160030, India; b Department of Orthopaedics, Max Super Speciality Hospital, Sahibzada Ajit Singh Nagar, Mohali, 160062, India

**Keywords:** Acetabulum, Elderly, Fracture, Osteoporosis, Hip, Arthroplasty

## Abstract

Fractures of the acetabulum in elderly patients were often caused by low energy trauma. Fractures involving anterior column are more common and often associated with impaction and comminution. Osteoporosis further complicates the management. Percutaneous fracture fixation has low morbidity but it is a technically demanding procedure. Open reduction and fracture fixation is done with or without simultaneous total hip replacement. Delayed total hip replacement is considered in posttraumatic arthritis patients. Patients with minimal displaced fractures, associated both-column fractures with secondary congruence of joint and patients who are medically unfit for surgery can be managed non-operatively. Whatever be the method of management, these elderly patients should be mobilised as early as possible.

## Incidence and mechanism

Long life expectancy in present times has led to increased aging population worldwide. Mean age of patients having acetabulum fractures as well as the proportion of elderly patients among acetabulum fractures are also rising.^1^ Although acetabulum fracture is a result of high energy trauma in young patients, a low energy fall can lead to acetabulum fractures in elderly because of poor bone quality. A low energy fall is reported as a cause of acetabulum fracture in elderly patients in up to 70% of cases.^2^ A fall which results an impact on greater trochanter causes anteromedial force on acetabulum. This anteromedial force typically causes fracture of anterior column along with quadrilateral plate with associated anteromedial displacement of femoral head. Poor bone quality results in articular impaction on superomedial aspect of acetabulum by anteromedially displaced head which is seen as “Gull Sign” on radiographs. At times there may be articular impaction on femoral head side by the intact acetabulum. Overall, the comminution and impaction are more common in elderly patients with poor bone quality than young patients.^1^

## Radiographic evaluation

The standard antero-posterior (AP) view along with iliac and obturator oblique Judet views of pelvis are required at minimal. CT scan with three-dimensional reconstruction helps in better understanding of fracture morphology and planning of surgery. Ferguson et al.^1^ reported that CT scan was helpful in diagnosing bone comminution and impaction which was not visible on plane radiographs. Fracture of the acetabulum in elderly patients may be missed easily.^3^ These occult fractures not seen on routine radiographs may be diagnosed on MRI scan similar to other hip fractures in elderly.^4^

The most common fracture pattern is associated both-column fracture.^1^^,^^5^ Fractures involving anterior column or anterior wall constitute 50%–60% of these fractures.^1^^,^^5^ Though the fractures are classified in the same manner as done in younger patients, some findings are more common and important to note. “Gull Sign” visible on AP view of pelvic radiograph signifies articular impaction on superomedial acetabulum by anteromedial migrated femoral head. Femoral head injury is also common and reported in up to 20% of cases.^1^ Marginal impaction and comminution are more common in elderly patients because of osteoporotic bone and should not be missed. Pre-existing arthritic changes if present should be made note as the patient may be a candidate for primary hip replacement surgery.

Radiographs and CT scans should also be used to evaluate for planning surgery. Methods of fracture reduction, plate and screw positioning, disimpaction of marginal impaction and need of bone grafting should be planned well in advance. If joint replacement is in consideration, the size and position of prosthesis and cementing options should be planned.

## Treatment options

Elderly patients with comorbid conditions should be identified and managed by concerned specialists as a team approach on urgent basis. Elderly trauma patients have mortality rate up to 3 times more than younger patients and it has been reported that the combined approach by orthopaedic and geriatric team improves outcome in elderly hip fracture patients.^6^^,^^7^ Osteoporotic bone, articular impaction, fracture comminution, pre-existing hip joint arthritis and multiple comorbidities further complicated the management.^8^ Whatever the treatment modality, it should be individualized according to fracture characteristic, patient's physiologic age and functional demand along with goal of early ambulation.^9^

## Non-operative management

There are no specific indications available for non-operative acetabulum fractures in elderly. However, because of low functional demand and multiple comorbidities in elderly patients, non-operative treatment may be an option. Spencer et al.^10^ retrospectively reviewed 25 patients with mean age of 74 (range 65–95) years where fracture acetabulum was managed non-operatively. There were 2 deaths and 7 patients with unacceptable results. Firoozabadi et al.^2^ reported 44% mortality rate in elderly patients managed non-operatively for acetabulum fractures as compare to 12% in those who managed surgically. However, the authors stated that this may be due to the fact that morbidly sick patients were less likely to have surgical intervention. Though historically the results of non-operative management in these patients are poor, non-operative management can be considered in certain conditions.

Criteria for non-operative management in adult patients also can be used for elderly patients. Minimally displaced fractures can be managed non-operatively. Also associated both-column fracture pattern exhibiting secondary congruence of hip joint can be managed without surgery. Tomgren et al.^8^ stated that elderly patients can be managed non-operatively if femur head is congruent with acetabulum without traction, fracture displacement is < 5 mm in weight bearing dome and in cases of posterior wall fractures involving <50% articular surface, hip joint is stable during dynamic stress under anaesthesia. The patients who are medically unfit for surgery should also be managed non-operatively. Patients with low functional demand who are ambulatory within home only may be a candidate for non-operative treatment, though the possibility of complications related to prolonged recumbency is high. If managed non-operatively early mobilisation should be encouraged in these patients.

### Open reduction and internal fixation (ORIF)

ORIF of acetabulum fractures in elderly patients is a topic of debate and the results are inconsistent in different studies. Bone quality and possibility of acceptable reduction should be considered before attempting open reduction and fixation in elderly patients. Tannast et al.^11^ reviewed 816 patients who were treated with open reduction of acetabulum fracture. The authors reported that increasing age was a negative predictor of survivorship of hip joint. The authors also reported that anterior wall fractures had poor outcome and this might be due to higher proportion of elderly patients in this subset of patients.

Matta^12^ reviewed 255 acetabular fractures fixed by ORIF with the mean follow-up of 6 years.^12^ The author concluded that fracture reduction was the key for good clinical results and an increasing age of patient adversely affects the reduction as well as clinical results. Anglen et al.^13^ reported anatomical reduction on post-operative radiographs in 61% of their cases after ORIF of acetabulum fractures in patients with age more than 60 years. Though some authors have reported poor outcome after acetabulum fractures in elderly patients, reasonable outcome has been reported in other 7 patients after imperfect fracture reduction. Miller et al.^14^ analysed post-operative fracture reduction after ORIF in 45 patients with mean age of 67 (range 59–82) years.^14^ The authors achieved anatomical reduction in 26 patients only on post-operative radiographs, while none of the patients had anatomical reduction on CT scan. However, clinical results were not found to be correlated with radiological reduction at follow-up of average 72.4 months. Similarly, Archdeacon et al.^15^ concluded in their study that reasonable functional results can be achieved even without anatomical reduction of acetabulum fractures in elderly patients. Helfet el al.^16^ analysed the results of 18 patients with average age of 67 (range 60–81) years. The authors concluded that ORIF of fracture acetabulum in elderly patients can yield good outcomes.

“Gull Sign” has been considered to be a predictor for poor results in elderly. Anglenet al.^13^ analysed patients with age more than 60 years after ORIF of acetabulum fracture and concluded that patients having “Gull Sign” on radiograph did not benefit from surgery. However, the authors did not addressed the osteochondral impaction in any of their patient and this may be the reason of failure. Casstevens et al.^17^ was firstly described a novel technique to reduce and buttress the articular impaction of superomedial dome of fractures acetabulum using modified Stoppa approach. Laflamme et al.^18^ used modified Stoppa approach to access the impacted superomedial part of fractured acetabulum in 9 elderly patients with an average age of 64.3 years. The impacted superomedial osteochondral acetabulum was disimpacted and supported with 3.5 mm cortical raft screw after filling the void with bone graft. The authors reported that superomedial impaction leaving less than 1 cm intact lateral margin led to medialisation of femoral head.

Fracture of quadrilateral plate is also more common in elderly than in younger patients due to their typical mechanism of injury and poor bone quality. Different authors have described techniques to stabilise the quadrilateral plate fracture associated with acetabular fractures.^19^, ^20^, ^21^, ^22^, ^23^ Laflamme et al.^24^ analysed the functional results of 19 patients with average age of 64.3 years having acetabulum fractures involving quadrilateral plate. Buttress plating of quadrilateral plate was done using modified Stoppa approach. The authors did not address “Gull Sign” and it was correlated with mal-reduction and later on arthroplasty. Archdeacon et al.^15^ fixed quadrilateral plate fractures with buttress plate in patients with age over 70 years and reported reasonable functional outcome even with less than anatomical reduction. Modified Stoppa approach has an advantage of being a less morbid approach than ilio-inguinal approach for anterior acetabulum fracture fixation.^23^ Also this approach provides a good access to acetabulum from inside the pelvis for reduction and fixation of quadrilateral plate as well as impacted superomedial portion of acetabulum.^18^^,^^24^ This makes modified Stoppa approach a preferable approach for anterior acetabulum fracture fixation in elderly patients.

Sen et al.^25^ in their literature review studied various anatomic plates, including a newly designed anatomical quadrilateral plate, to fix fractured quadrilateral plate. The authors argued that none of the previously available implants supports the pelvic brim at ilio-pectineal line where the vector of fracture causing force primarily hits. This makes fracture fixation difficult especially in elderly patients with osteoporotic bones. The authors suggest that anatomical quadrilateral plate neutralises the force vector at ilio-pectineal line and is likely to be useful in acetabulum fractures in elderly.

## Minimally invasive methods

Minimal invasive fracture fixation has advantage of less blood loss, less chances of infection and quicker recovery as compare to ORIF. Gay et al.^26^ in 1992 was the first to describe percutaneous fixation of acetabulum. The authors fixed acetabulum fracture percutaneously under CT guidance in 6 patients. Starr et al.^27^ in 1998 described technique for percutaneous fixation of acetabulum fractures under fluoroscopic imaging using 6.5 or 7.3 mm cannulated screws. Gary et al.^28^ analysed the rate of conversion to total hip arthroplasty after percutaneous fixation of displaced acetabulum fractures in patients with age over 60 years. The authors retrospectively reviewed 80 acetabulum fractures. In 20 of 80 fractures reduction was done percutaneously by using ball spike pusher or cob elevator under fluoroscopic guidance. In remaining 60 patients, minimal open reduction was done through 3 cm lateral window of ilio-inguinal approach. Seventy-five patients had adequate follow-up of mean 3.9 (range 0.5–11.9) years. There was no infection in any of the case and the mean blood loss was 69 mL. Total hip arthroplasty was done in 19 of 75 patients after mean 1.4 years of fracture fixation. The authors concluded that the rate of conversion to total hip arthroplasty after percutaneous fixation was comparable to open method of fracture fixation. The same group of patients were further followed at mean 6.8 years post-operatively for functional outcome.^29^ Thirty-five patients were analysed and it was concluded that functional outcome in elderly patients who were operated for acetabulum fracture with percutaneous fixation was not different compared with ORIF. Daurka et al.^5^ also in their systematic review reported reoperation rates similar to Gary et al.,^29^ when percutaneous fixation and ORIF were compared. The authors further concluded that the rate of mortality may not improve significantly. Recently, Ernstberger et al.^30^ compared ORIF, percutaneous and non-operative treatment methods for minimally displaced acetabular fractures in patients with age over 60 years. The percutaneous group had lesser blood loss, surgical time, hospital stay and non-surgical complications as compare to ORIF group. However, the surgical complications were comparable in both groups. Further the authors did not find any significant difference in quality of reduction between 2 groups.

Though minimal invasive fixation has its advantages, the procedure is technically demanding and the learning curve is steep. Though loss of reduction was reported by some authors, the more recent studies showed better results.^30^^,^^31^ Good understanding of pelvic anatomy and interpretation of intra-operative radiographic imaging is utmost important.

## Total hip arthroplasty

Literature regarding total hip replacement after acetabulum fracture specific to elderly patients is highly variable. In a review article of Makridis et al.^32^ reported that clinical results of total hip arthroplasty after acetabulum fractures were poorer in patients over 70 years of age as compare to younger patients and this was regardless of timing of surgery. However, the revision rates were higher in younger patients because of high level of activity as compare to elderly.^32^

## Delayed total hip arthroplasty

Delayed total hip arthroplasty is indicated in patients who develop post-traumatic arthritis after either ORIF or non-operatively managed fracture acetabulum. Though delayed reconstruction has advantage of defined bone stock with consolidated fracture, arthroplasty after non-operatively managed fractures have technically challenges of non-union or osteonecrosis of fracture fragments, bone loss and deformed acetabulum.^33^ Scarred tissue, risk of sciatic nerve injury, metalwork of previous fixation and heterotopic ossification poses additional problems in previously operated fracture acetabulum cases.^33^

Romness et al.^34^ reported that revision rate of delayed total hip replacement after fracture acetabulum was 17.2% in patients under 60 years of age as compare to 7.7% in patients with age over 60 years.^34^ The literature on delayed total hip arthroplasty after acetabulum fractures in elderly is scanty and more work need to be done in this specific area.

## Acute total hip arthroplasty

Main indications for acute total hip replacement after acetabulum fracture at any age are subchondral impaction on either acetabular or femoral head side, femur head fracture, severe intra articular comminution and pre-injury symptomatic hip arthritis.^35^ Old age is also considered as a poor prognostic factor after fracture acetabulum and acute total hip replacement. Tannast et al.^11^ in their review of 816 surgically treated acetabulum fractures concluded that increasing age was a negative predictor of survivorship of hip joint. Kreder et al.^36^ evaluated functional, clinical and radiological outcome of 128 patients after ORIF of acetabulum fracture. The authors concluded that anatomical reduction of fracture was not always sufficient for good functional outcome. The authors suggested acute fracture fixation and total hip replacement in patients with age over 50 years having marginal impaction and fracture comminution. Similarly in review article of De Bellis et al.^37^, they reported that old age was an indication for acute fracture fixation and simultaneously total hip replacement.

Lin et al.^38^ analysed 33 fracture acetabulum patients treated with combined ORIF and total hip replacement. The average age was 66 years with average follow-up of 67 months. Survival rate was 94% with good to excellent outcome in 93% of patients. The authors concluded that this combined approach was a safe and viable treatment option which can avoid 2 surgeries. Herscovici et al.^39^ analysed 22 patients with average age 75.3 years where combined fixation and total hip arthroplasty was done for fracture acetabulum.^39^ The authors also reported that results with ilio-inguinal approach were worse in these patients as compare to kocher-lagenbeck approach.

Rickman et al.^40^ emphasised that fracture acetabulum in elderly patients presents problems similar to proximal femur fractures in same age group and so early weight bearing is the key to maximise outcome.^40^ The authors treated 25 hips in 24 patients having low energy osteoporotic acetabular fractures with average age of 77 (range 63–90) years. Combined fracture fixation with total hip replacement was done and immediate weight bearing was started as post-operative protocol. One patient died and results were reported for 23 patients on average follow-up of 24 (range 8–38) months. There was no component migration and all 23 patients returned to pre-injury residential status by 12 weeks after injury.

Different techniques have been described for fracture fixation while doing acute simultaneous fixation and total hip replacement in fracture acetabulum.^41^, ^42^, ^43^, ^44^, ^45^ Mears et al.^41^ described for the first time the use of cables to fix the acetabulum fractures in such cases. The authors stated that the technique provided effective immobilisation of fracture and especially helpful in elderly patients with osteoporotic bones.^41^ Mouhsine et al.^42^ also fixed fracture acetabulum with cable wires for doing acute total hip replacement in 18 elderly patients with mean follow-up of 36 months. The authors concluded that the method provided good primary fixation and allowed early post-operative mobilisation. Chakravarty et al.^46^ described novel technique of treatment of acetabular fractures in elderly. The authors fixed 19 fractures using percutaneous screws and simultaneously total hip replacement was done. Though the blood loss was less as compare to previous available literature for combined fracture fixation and total hip replacement, the authors did not recommend this method for elderly patients due to significant complication rates. However, the authors also stated that the high complication rates may be due to confounding variables such as comorbid conditions.

Tidermark et al.^47^ reviewed retrospectively 10 patients with mean age of 73 (range 57–87) years where acute total hip replacement was done for fracture acetabulum. Reinforcement ring (Burch-Schneider antiprotrusion cage) was used to stabilise the acetabular cup rather than fixing the fracture. Bone graft from femur head was packed into the acetabulum and reinforcement ring was fixed to axial skeletal. Since the stability was due to the fixation of reinforcement ring to the axial skeleton, the authors excluded associated both-column fracture for this type of treatment method. Enocson et al.^48^ used similar technique to treat 15 patients with age more than 60 years. With 4 year follow-up the authors concluded that the method was safe with good clinical and radiological outcomes. Daurka et al.^5^ in their systematic review concluded that the results were conflicting when acute combined fracture fixation and total hip replacement was compared with delayed total hip replacement.

Kocher-langenbach approach as a single approach is sufficient to fix posterior acetabulum fractures as well as for total hip replacement in cases where simultaneously fracture fixation and hip replacement were planned. However in such patients with anterior acetabulum fracture, ilio-inguinal approach is not adequate to fix femoral component. Beaule et al.^49^ used Levine modification of smith-peterson approach to fix anterior acetabulum fractures and simultaneously total hip replacement.^49^ The advantages were single incision, less operating time and thus less morbidity.

Recently, Jauregui et al.^50^ did a meta-analysis of acute total hip replacement in patients with age of 60 years or more having acetabular fractures. The authors evaluated 21 studies with 430 acetabular fractures with mean follow-up of 44 months. Mean Harris hip score was 83.3 points and 20% patients reported various complications. The authors concluded that acute total hip replacement in acetabular fractures may have higher revision and complication rates as compare to primary total hip replacement but the results are comparable to those having primary fracture fixation followed by delayed hip replacement.

## Rehabilitation

Rehabilitation in elderly patients is always a concern. The patients should be mobilised as early as possible whatever the method of treatment. However there are no standard guidelines for rehabilitation after acetabulum fracture in elderly patients. Different authors have analysed these fractures for return to previous level of mobilisation. Walley et al.^51^ compared the outcome of operated and non-operated acetabular fractures in elderly patients and reported that 76% of operated patients were not returned to previous level of ambulation as compare to 71% of non-operated patients. Wollmerstädt et al.^52^ found that 65% of patients with fragility fracture acetabulum were able to return their home.^52^ The authors further reported that patients received operated treatment stayed longer in hospital than patients with non-operative treatment.

## Summary

Fracture acetabulum in elderly patients is a low energy trauma injury. Like in other fractures around hip the aim should be to mobilise the patient earliest possible. Minimally displaced fractures and associated both-column fractures with secondary congruence of hip joint can be managed non-operatively. ORIF is difficult due to poor bone quality in elderly patients. Articular impaction of superomedial acetabulum and quadrilateral plate fractures are common and have poor prognosis. In such cases acute total hip replacement along with fracture fixation may be a good option. In cases of post-traumatic arthritis, either after fracture fixation or in non-operatively managed patients, delayed total hip replacement can be considered. Overall the management of elderly patients with fracture acetabulum is a challenge.

## Funding

Nil.

## Ethical statement

Not applicable.

## Declaration of competing interest

The authors declare that there is no conflict of interest.

## Author contributions

Ashwani Soni wrote the article. Ravi Gupta conceptualised the idea. Ramesh Sen made correction and finalised the manuscript.

## References

[1] Ferguson T.A., Patel R., Bhandari M. (2010). Fractures of the acetabulum in patients aged 60 years and older: an epidemiological and radiological study. J Bone Joint Surg Br.

[2] Firoozabadi R., Cross W.W., Krieg J.C. (2017). Acetabular fractures in the senior population- epidemiology, mortality and treatments. Arch Bone Jt Surg.

[3] Tornkvist H., Schatzker J. (1993). Acetabular fractures in the elderly: an easily missed diagnosis. J Orthop Trauma.

[4] Iwata T., Nozawa S., Dohjima T. (2012). The value of T1-weighted coronal MRI scans in diagnosing occult fracture of the hip. J Bone Joint Surg Br.

[5] Daurka J.S., Pastides P.S., Lewis A. (2014). Acetabular fractures in patients aged 55 > years: a systematic review of the literature. Bone Joint Lett J.

[6] Keller J.M., Sciadini M.F., Sinclair E. (2012). Geriatric trauma: demographics, injuries, and mortality. J Orthop Trauma.

[7] Friedman S.M., Mendelson D.A., Bingham K.W. (2009). Impact of a comanaged Geriatric Fracture Center on short-term hip fracture outcomes. Arch Intern Med.

[8] Tomgren T.R., Szatkowski J.P., Perez E.A. (2011). Geriatric orthopaedics: acetabular fractures in elderly. Current Orthopaedic Practice.

[9] Henry P.D., Kreder H.J., Jenkinson R.J. (2013). The osteoporotic acetabular fracture. Orthop Clin North Am.

[10] Spencer R.F. (1989). Acetabular fractures in older patients. J Bone Joint Surg Br.

[11] Tannast M., Najibi S., Matta J.M. (2012). Two to twenty-year survivorship of the hip in 810 patients with operatively treated acetabular fractures. J Bone Joint Surg Am.

[12] Matta J.M. (1996). Fractures of the acetabulum: accuracy of reduction and clinical results in patients managed operatively within three weeks after the injury. J Bone Joint Surg Am.

[13] Anglen J.O., Burd T.A., Hendricks K.J. (2003). The "Gull Sign": a harbinger of failure for internal fixation of geriatric acetabular fractures. J Orthop Trauma.

[14] Miller A.N., Prasarn M.L., Lorich D.G. (2010). The radiological evaluation of acetabular fractures in the elderly. J Bone Joint Surg Br.

[15] Archdeacon M.T., Kazemi N., Collinge C. (2013). Treatment of protrusion fractures of the acetabulum in patients 70 years and older. J Orthop Trauma.

[16] Helfet D.L., Borrelli J., DiPasquale T. (1992). Stabilization of acetabular fractures in elderly patients. J Bone Joint Surg Am.

[17] Casstevens C., Archdeacon M.T., dʼHeurleA (2014). Intrapelvic reduction and buttress screw stabilization of dome impaction of the acetabulum: a technical trick. J Orthop Trauma.

[18] Laflamme G.Y., Hebert-Davies J. (2014). Direct reduction technique for superomedial dome impaction in geriatric acetabular fractures. J Orthop Trauma.

[19] Letournel E., Judet R. (1993).

[20] Farid Y.R. (2010). Cerclage wire-plate composite for fixation of quadrilateral plate fractures of the acetabulum: a checkrein and pulley technique. J Orthop Trauma.

[21] Culemann U., Marintschev I., Gras F. (2011). Infraacetabular corridor–technical tip for an additional screw placement to increase the fixation strength of acetabular fractures. J Trauma.

[22] Abo-Elsoud M., Radwan Y.A., Gobba M. (2014). Short segment fixation through a limited ilioinguinal approach for treating anterior acetabular fractures: a historical-control study. Int Orthop.

[23] Soni A., Gupta R., Sen R. (2019). Modified Stoppa approach for acetabulum fracture: a review. Rev Bras Ortop (Sao Paulo)..

[24] Laflamme G.Y., Hebert-Davies J., Rouleau D. (2011). Internal fixation of osteopenicacetabular fractures involving the quadrilateral plate. Injury.

[25] Sen R.K., Saini G., Kadam S. (2020). Anatomical quadrilateral plate for acetabulum fractures involving quadrilateral surface: a review. J Clin Orthop Trauma.

[26] Gay S.B., Sistrom C., Wang G.J. (1992). Percutaneous screw fixation of acetabular fractures with CT guidance: preliminary results of a new technique. AJR Am J Roentgenol.

[27] Starr A.J., Reinert C.M., Jones A.L. (1998). Percutaneous fixation of the columns of the acetabulum: a new technique. J Orthop Trauma.

[28] Gary J.L., Lefaivre K.A., Gerold F. (2011). Survivorship of the native hip joint after percutaneous repair of acetabular fractures in the elderly. Injury.

[29] Gary J.L., VanHal M., Gibbons S.D. (2012). Functional outcomes in elderly patients with acetabular fractures treated with minimally invasive reduction and percutaneous fixation. J Orthop Trauma.

[30] Ernstberger H., Pieroh P., Höch A. (2021). Minimally displaced acetabulum fractures in geriatric patients: a comparison of open, percutaneous and non-operative treatment from the German Pelvic Injury Register data. Eur J Trauma Emerg Surg.

[31] Starr A.J., Jones A.L., Reinert C.M. (2001). Preliminary results and complications following limited open reduction and percutaneous screw fixation of displaced fractures of the acetabulum. Injury.

[32] Makridis K.G., Obakponovwe O., Bobak P. (2014). Total hip arthroplasty after acetabular fracture: incidence of complications, reoperation rates and functional outcomes: evidence today. J Arthroplasty.

[33] Sierra R.J., Mabry T.M., Sems S.A. (2013). Acetabular fractures: the role of total hip replacement. Bone Joint Lett J.

[34] Romness D.W., Lewallen D.G. (1990). Total hip arthroplasty after fracture of the acetabulum. Long-term results. J Bone Joint Surg Br.

[35] Mears D.C., Velyvis J.H. (2002). Acute total hip arthroplasty for selected displaced acetabular fractures: two to twelve-year results. J Bone Joint Surg Am.

[36] Kreder H.J., Rozen N., Borkhoff C.M. (2006). Determinants of functional outcome after simple and complex acetabular fractures involving the posterior wall. J Bone Joint Surg Br.

[37] De Bellis U.G., Legnani C., Calori G.M. (2014). Acute total hip replacement for acetabular fractures: a systematic review of the literature. Injury.

[38] Lin C., Caron J., Schmidt A.H. (2015). Functional outcomes after total hip arthroplasty for the acute management of acetabular fractures: 1- to 14-year follow-up. J Orthop Trauma.

[39] Herscovici D., Lindvall E., Bolhofner B. (2010). The combined hip procedure: open reduction internal fixation combined with total hip arthroplasty for the management of acetabular fractures in the elderly. J Orthop Trauma.

[40] Rickman M., Young J., Trompeter A. (2014). Managing acetabular fractures in the elderly with fixation and primary arthroplasty: aiming for early weight bearing. Clin Orthop Relat Res.

[41] Mears D.C., Shirahama M. (1998). Stabilization of an acetabular fracture with cables for acute total hip arthroplasty. J Arthroplasty.

[42] Mouhsine E., Garofalo R., Borens O. (2002). Acute total hip arthroplasty for acetabular fractures in the elderly: 11 patients followed for 2 years. Acta Orthop Scand.

[43] Manson T.T. (2020). Open reduction and internal fixation plus total hip arthroplasty for the acute treatment of older patients with acetabular fracture: surgical techniques. Orthop Clin North Am.

[44] Lont T., Nieminen J., Reito A. (2019). Total hip arthroplasty, combined with a reinforcement ring and posterior column plating for acetabular fractures in elderly patients: good outcome in 34 patients. Acta Orthop.

[45] Boelch S.P., Jordan M.C., Meffert R.H. (2017). Comparison of open reduction and internal fixation and primary total hip replacement for osteoporotic acetabular fractures: a retrospective clinical study. Int Orthop.

[46] Chakravarty R., Toossi N., Katsman A. (2014). Percutaneous column fixation and total hip arthroplasty for the treatment of acute acetabular fracture in the elderly. J Arthroplasty.

[47] Tidermark J., Blomfeldt R., Ponzer S. (2003). Primary total hip arthroplasty with a Burch–Schneider antiprotrusion cage and autologous bone grafting for acetabular fractures in elderly patients. J Orthop Trauma.

[48] Enocson A., Blomfeldt R. (2014). Acetabular fractures in the elderly treated with a primary Burch-Schneider reinforcement ring, autologous bone graft, and a total hip arthroplasty: a prospective study with a 4-year follow-up. J Orthop Trauma.

[49] Beaulé P.E., Griffin D.B., Matta J.M. (2004). The Levine anterior approach for total hip replacement as the treatment for an acute acetabular fracture. J Orthop Trauma.

[50] Jauregui J.J., Weir T.B., Chen J.F. (2020). Acute total hip arthroplasty for older patients with acetabular fractures: a meta-analysis. J Clin Orthop Trauma.

[51] Walley K.C., Appleton P.T., Rodriguez E.K. (2017). Comparison of outcomes of operative versus non-operative treatment of acetabular fractures in the elderly and severely comorbid patient. Eur J Orthop Surg Traumatol.

[52] Wollmerstädt J., Pieroh P., Schneider I. (2020). Mortality, complications and long-term functional outcome in elderly patients with fragility fractures of the acetabulum. BMC Geriatr.

